# Amino acid metabolism in primary bone sarcomas

**DOI:** 10.3389/fonc.2022.1001318

**Published:** 2022-10-05

**Authors:** Jennifer A. Jiménez, Elizabeth R. Lawlor, Costas A. Lyssiotis

**Affiliations:** ^1^ Department of Molecular & Integrative Physiology, University of Michigan Medical School, Ann Arbor, MI, United States; ^2^ Rogel Cancer Center, University of Michigan Medical School, Ann Arbor, MI, United States; ^3^ Department of Pediatrics, University of Washington, Seattle, WA, United States; ^4^ Seattle Children’s Research Institute, Seattle, WA, United States; ^5^ Department of Internal Medicine, Division of Gastroenterology and Hepatology, University of Michigan Medical School, Ann Arbor, MI, United States

**Keywords:** tumor metabolism, sarcoma, Ewing sarcoma, amino acid metabolism, osteoclast, osteoblast

## Abstract

Primary bone sarcomas, including osteosarcoma (OS) and Ewing sarcoma (ES), are aggressive tumors with peak incidence in childhood and adolescence. The intense standard treatment for these patients consists of combined surgery and/or radiation and maximal doses of chemotherapy; a regimen that has not seen improvement in decades. Like other tumor types, ES and OS are characterized by dysregulated cellular metabolism and a rewiring of metabolic pathways to support the biosynthetic demands of malignant growth. Not only are cancer cells characterized by Warburg metabolism, or aerobic glycolysis, but emerging work has revealed a dependence on amino acid metabolism. Aside from incorporation into proteins, amino acids serve critical functions in redox balance, energy homeostasis, and epigenetic maintenance. In this review, we summarize current studies describing the amino acid metabolic requirements of primary bone sarcomas, focusing on OS and ES, and compare these dependencies in the normal bone and malignant tumor contexts. We also examine insights that can be gleaned from other cancers to better understand differential metabolic susceptibilities between primary and metastatic tumor microenvironments. Lastly, we discuss potential metabolic vulnerabilities that may be exploited therapeutically and provide better-targeted treatments to improve the current standard of care.

## 1 Introduction: Bone sarcoma and metabolism

Primary bone cancers encompass approximately 0.2% of all malignant tumors, with osteosarcoma (OS), Ewing sarcoma (ES), and chondrosarcoma representing 90-95% of these diagnoses (1). ES and OS are predominately cancers of childhood and adolescence. While survival rates for other childhood malignancies have improved steadily in recent decades, the five-year survival rate for ES and OS patients presenting with metastatic disease at diagnosis has not improved and remains near 30% (2). Furthermore, for patients with localized disease, the standard of care for these highly aggressive bone tumors consists of high-dose chemotherapy, surgery, and radiation. This leads to significant long-term toxicities that contribute to both decreased life expectancy and diminished quality of life (2).

Throughout life, normal bone metabolism and growth are dependent on maintaining a balance between the constant and coincident processes of bone formation and bone resorption. Disruption to this balance in bone remodeling often occurs during normal aging when bone resorption can exceed bone formation, resulting in osteoporosis (3). In contrast, during adolescence, the time at which ES and OS most commonly arise, rapid growth requires that bone formation, or osteogenesis, exceed the level of bone resorption. Normal bone remodeling is a high-energy process that is dependent on cellular metabolic processes, and the central cellular mediators that carry out the processes of bone building and bone destruction are osteoblasts and osteoclasts, respectively. While it is appreciated that the skeleton is a metabolically active organ, and studies investigating the bioenergetic properties of bone can be traced back over 60 years (4–6), until recently, the metabolic dependencies of bone homeostasis had been relatively unexplored.

Dysregulated metabolism is recognized as a hallmark of cancer (7), where the rewiring of metabolic pathways is required for supporting the biosynthetic demands of highly proliferative malignant cells (8). Early interest in the field of cancer metabolism focused on glucose metabolism, with more recent work revealing the importance of amino acids in cancer progression (9). Amino acids play a central role, not only in protein synthesis, but also energy production, nucleotide synthesis, and maintenance of redox homeostasis (10, 11). More importantly, advancements have been made in the feasibility of targeting of amino acid metabolic pathways that are rewired in cancer, which may provide a therapeutic vulnerability (11). This has led to the preclinical and clinical development of therapeutic drugs, such as amino acid degrading enzymes, amino acid transporter inhibitors, and those that target *de novo* amino acid biosynthetic pathways. However, determination and therapeutic exploitation of metabolic vulnerabilities in primary bone tumors has lagged relative to advances for other tumor types.

In this review, we provide an overview of the amino acid metabolic pathways that are central in the “normal” bone microenvironment, primarily focused on the bioenergetics of the osteoblasts and osteoclasts that maintain bone homeostasis. We further contrast amino acid dependencies in the normal bone environment with the amino acid requirements of primary bone malignancies, focused mainly on OS and ES. Lastly, we provide a summary of therapeutically targetable pathways that are currently under study.

## 2 Metabolic rewiring in cancer

### 2.1 Amino acid metabolism

**Figure 1:**
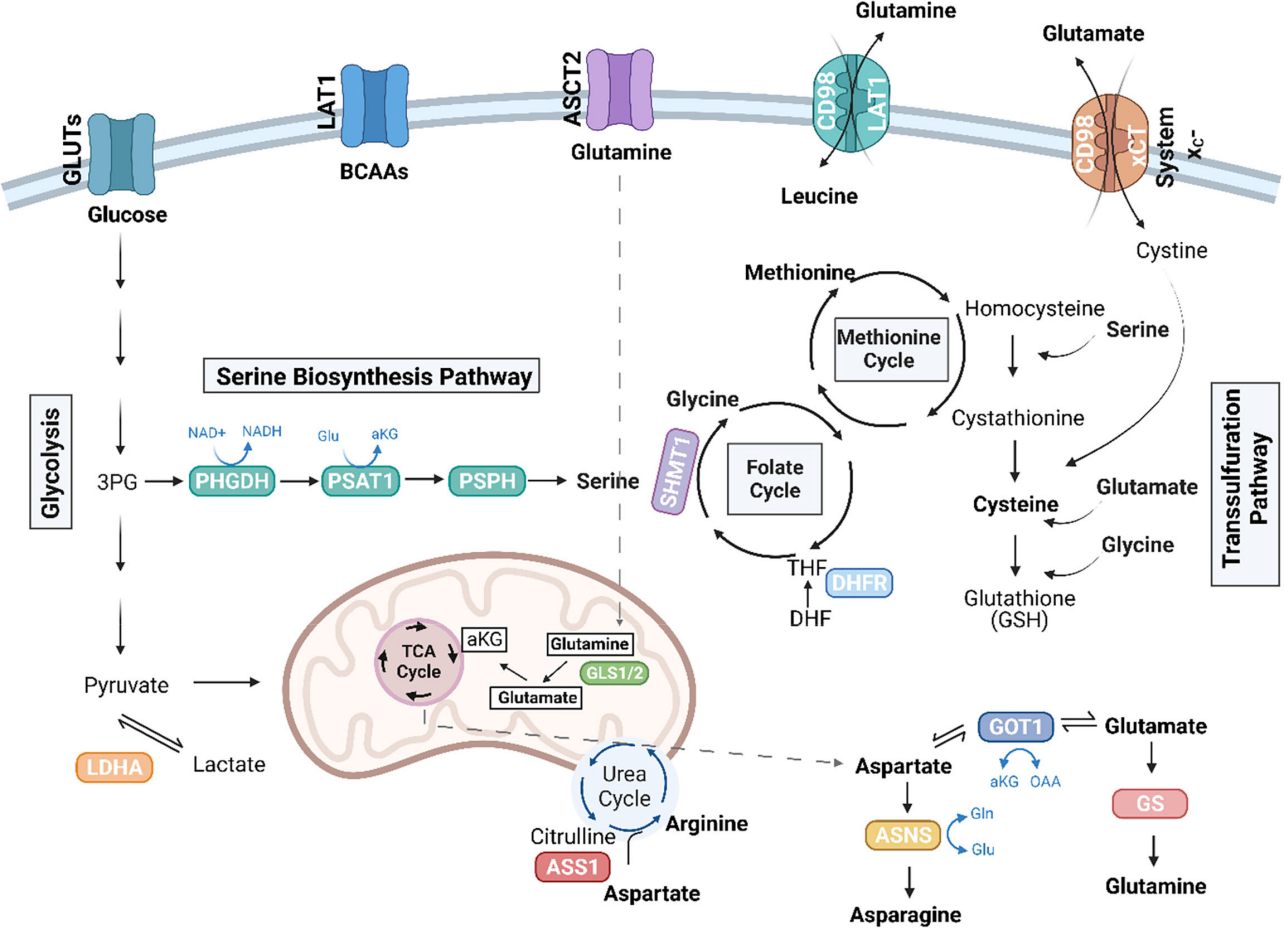
Amino acid pathways rewired in cancer. Illustration depicting amino acid pathways linked in an intricate metabolic network. Essential amino acids, including the branched chain amino acids (BCAAs), leucine, isoleucine, and valine, are imported from the extracellular environment, in part via the LAT1 (*SLC7A5*) transporter. Glutamine (Gln) can be conditionally essential and is imported through ASCT2 (*SLC1A5*). Glutaminase enzymes (GLS1/2) convert glutamine to glutamate (Glu), which can further be converted to alpha-ketoglutarate (αKG) to enter the TCA cycle. The TCA cycle also generates intermediates, which give rise to non-essential amino acids, including aspartate and arginine. The *de novo* synthesis of arginine involves the conversion of citrulline to aspartate by argininosuccinate synthetase 1 (ASS1). TCA-cycle derived aspartate is converted to asparagine via ASNS, and GOT1 can convert aspartate to glutamate, which is further converted to glutamine by glutamine synthetase (GS). Serine, another non-essential amino acid, can be imported or synthesized *de novo* in the serine biosynthesis pathway (SBP), which diverts the 3P-glycerate (3PG) intermediate of glycolysis to generate serine via the enzymes, phosphoglycerate dehydrogenase (PHGDH), phosphoserine aminotransferase (PSAT1), and phosphoserine phosphatase (PSPH). Serine can further be converted to glycine by serine hydroxymethyltransferase (SHMT) (cytosolic SHMT1 isozyme shown above) to generate one-carbon units to fuel the folate cycle and the methionine cycles. Dihydrofolate reductase (DHFR) in the folate cycle catalyzes the reduction of dihydrofolate to tetrahydrofolate (THF). Cysteine is another non-essential amino acid that can be synthesized in the transsulfuration pathway or imported as cystine via the cystine/glutamate antiporter, system x_C_-. The SBP, transsulfuration pathway, along with ASS1 and glutamine metabolism have been implicated in sarcoma biology. Figure was created with BioRender.com.

Cellular metabolism describes the series of biochemical reactions that generate energy and biomolecules needed to sustain homeostasis and function. The balance between catabolic and anabolic processes allows cells to both harness and utilize energy, as well as synthesize and break down macromolecules as appropriate to sustain growth. As an example, the catabolism of glucose provides a major energy source for the generation of adenosine triphosphate (ATP), a principal energy currency for the maintenance of cellular homeostasis, through glycolysis and mitochondrial oxidative phosphorylation (OXPHOS) (**Figure 1**).

Apart from glucose, proliferating cells also exhibit a significant demand for amino acids, which, in part, are required to support protein synthesis. Essential amino acids, including the branched chain amino acids (BCAAs) leucine, isoleucine, and valine, cannot be synthesized within the cell and instead require import from the extracellular environment. Non-essential amino acids, including the amino acids glutamine, glutamate, alanine, proline, and aspartate, on the other hand, can by synthesized *de novo* providing cells with more flexibility when these amino acids become scarce (**Figure 1**). Aside from their proteinogenic function, amino acids have also been shown to play a role in energy production. Glutamine, the most abundant amino acid in circulation, can sustain the TCA cycle (**Figure 1**). Through glutaminolysis, the enzyme glutaminase (GLS) converts glutamine to glutamate, which can then be converted to alpha-ketoglutarate (αKG) to enter the TCA cycle. Glutamine also serves as a precursor for other non-essential amino acids, including aspartate, alanine, arginine, and proline. Additionally, non-essential amino acids participate in the regulation of signaling pathways and maintenance of cellular redox homeostasis; for example cysteine, glycine, and glutamate constitute the tripeptide and major antioxidant glutathione (GSH) (11).

### 2.2 Amino acid metabolism in cancer

The observation that cancer cells exhibit a rewiring of metabolic processes can be traced back to the work of Otto Warburg, with his finding that cancer cells preferentially use glycolysis, even in the presence of oxygen. This process is termed aerobic glycolysis and is known colloquially as the Warburg effect (12). In tumors and proliferative cells, glucose uptake is high. Aerobic glycolysis provides glycolytic intermediates necessary for anabolic reactions that generate building blocks to sustain biomass accumulation in cells (13). This metabolism is reenforced by glutamine, which is used to fuel the TCA cycle for energy production and also supplies carbon and nitrogen for the generation of biosynthetic precursors such as nucleotides, non-essential amino acids, and fatty acids (14, 15).

Beyond glucose and glutamine, cancers often become auxotrophic for various nutrients. This includes a dependence on an exogenous supply of non-essential amino acids, due to the loss of expression of key enzymes necessary for the synthesis of that amino acid, such as in the case of asparagine, arginine, and glutamine (11, 16, 17). In fact, one of the most successful amino acid-directed therapies targets the *de novo* synthesis of asparagine. It was found in the 1970s that the use of bacterial asparaginase (ASNase) could cure children with pediatric acute lymphoblastic leukemia (ALL) when used as a single agent or as a combination therapy, due to low expression of asparagine synthetase (ASNS) (**Figure 1**), the enzyme that converts aspartate to asparagine in leukemic blasts (18).

Cancers also depend on other *de novo* amino acid biosynthetic pathways and exhibit upregulated expression of enzymes in these pathways. For example, expression of phosphoglycerate dehydrogenase (PHGDH), the rate-limiting enzyme in the serine biosynthetic pathway (SBP), is upregulated in breast cancer and melanoma due to genomic amplification of the *PHGDH* locus. Further, this is essential for sustaining oncogenic growth (19, 20). Studies have demonstrated that the effect of inhibition of PHGDH in cancer cells cannot be fully rescued by extracellular serine alone, suggesting that these tumors depend on other intermediate products of the SBP (**Figure 1**) (20). A comprehensive review on amino acid metabolism in cancer, more broadly, is beyond the scope of the current review, and readers are directed to prior published reviews for recent summaries of this complex field of study (9, 10, 21, 22). While research is growing in this area, the investigation of amino acid requirements in primary bone sarcomas has just recently received due attention, and these new findings are detailed in the sections that follow.

## 3 Metabolism in normal bone homeostasis

### 3.1 Cycle of bone remodeling

**Figure 2:**
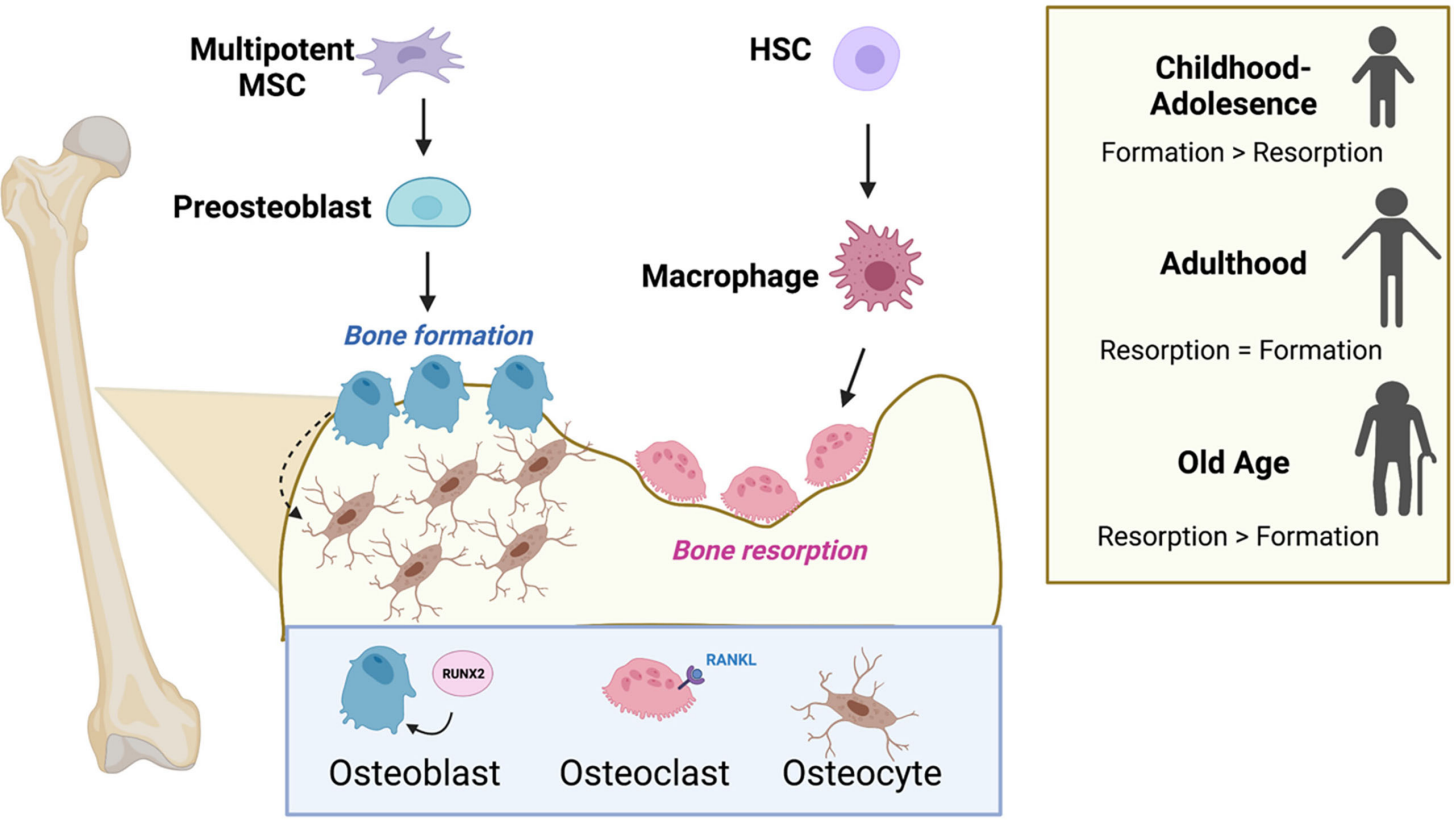
Osteoblasts, osteoclasts, and the cycle of bone remodeling. Bone remodeling balances the competing actions of the bone-building osteoblasts and the osteoclasts that promote bone resorption. Osteoblasts and osteocytes originate from common precursor bone marrow mesenchymal stem cells (MSCs), while osteoclasts are multinucleated and originate from hematopoietic stem cells (HSCs) in the bone marrow. Successful differentiation of progenitor cells into osteoblasts and osteoclasts requires the transcription factor, RUNX2 and RANK-ligand (RANKL), respectively. The level of bone formation versus bone resorption is dynamic and changes with age. Formation exceeds resorption during adolescence, and the scale tips the opposite direction favoring resorption in old age. Figure was created with BioRender.com.

The bone is a dynamic organ, with processes that maintain high turnover for constant bone remodeling. These cycles of building and destroying bone are commonly referred to as bone formation and bone resorption, respectively. The biological process of bone remodeling is required to maintain proper calcium homeostasis, respond to injury, and promote growth and development (23). In children, the rate of bone formation exceeds the level of bone resorption, supporting the growth of bone through adolescence. These two processes achieve a balance in adolescence, and eventually the scale tips toward a net loss of bone, which is associated with ageing (**Figure 2**). Defects in this cycle of bone turnover can contribute to diseases of bone such as osteoporosis, Paget’s disease of bone, osteogenesis imperfecta, and cancer, all of which interfere with the body’s normal bone-recycling processes (3, 24).

The chief cell types regulating bone homeostasis are the bone-building osteoblasts and the bone-destroying osteoclasts, which together make up ~5% of cells in the bone. Osteoblasts and osteocytes originate from common precursor bone marrow mesenchymal stem cells (23). Osteoclasts are multinucleated cells that originate from hematopoietic progenitors in the bone marrow and result from the fusion of myeloid lineage monocytes (osteoclast precursor cells) (**Figure 2**). The cycle of bone remodeling consists of two main phases, the formation of bone, whereby osteoblasts function to produce and secrete alpha-1 type-1 collagen and mineralize bone; and bone resorption, where osteoclasts residing in resorption bays digest bone by secreting proteases and acids to promote bone degradation (3). Osteocytes are terminally differentiated osteoblasts consisting of 90-95% of bone and are the longest living bone cells. They are encased in the matrix of mature bone and function by secreting matrix proteins and help to regulate osteoblast and osteoclast activity.

The complex signaling and molecular mechanisms that govern bone remodeling have been extensively studied. In brief, successful differentiation of progenitor cells into osteoblasts and osteoclasts is controlled, respectively, by the transcription factor RUNX2 (25) and the cytokine RANK-ligand (RANKL), a member of the tumor necrosis factor (TNF) superfamily (26) (**Figure 2**). Osteoblast function is regulated by numerous autocrine and paracrine signals. In particular, osteoblasts respond to a range of stimuli, including insulin-growth factors (IGF) (27), platelet-derived growth factor (PDGF) (28), TGF-β (29), and bone morphogenetic proteins (BMP) (30), as well as numerous classical hormones such as parathyroid and thyroid hormones (31). Similarly, osteoclast function is regulated by hormones including calcitonin (32), thyroid and parathyroid hormones (33, 34), IGF-1 (35), and PDGF (36). Osteoclasts and osteoblasts are the most metabolically active cells in the normal bone environment, but little is known about the bioenergetic properties of osteocytes. Emerging work suggests that osteoblasts and osteoclasts must acquire the proper nutrients and amino acids to sustain their differentiation and execute their proper functions in orchestration of bone remodeling. In the sections that follow, we describe the cell-type specific metabolic programs operative for osteoblast and osteoclast function.

### 3.2 Energy metabolism of the osteoblast

**Figure 3:**
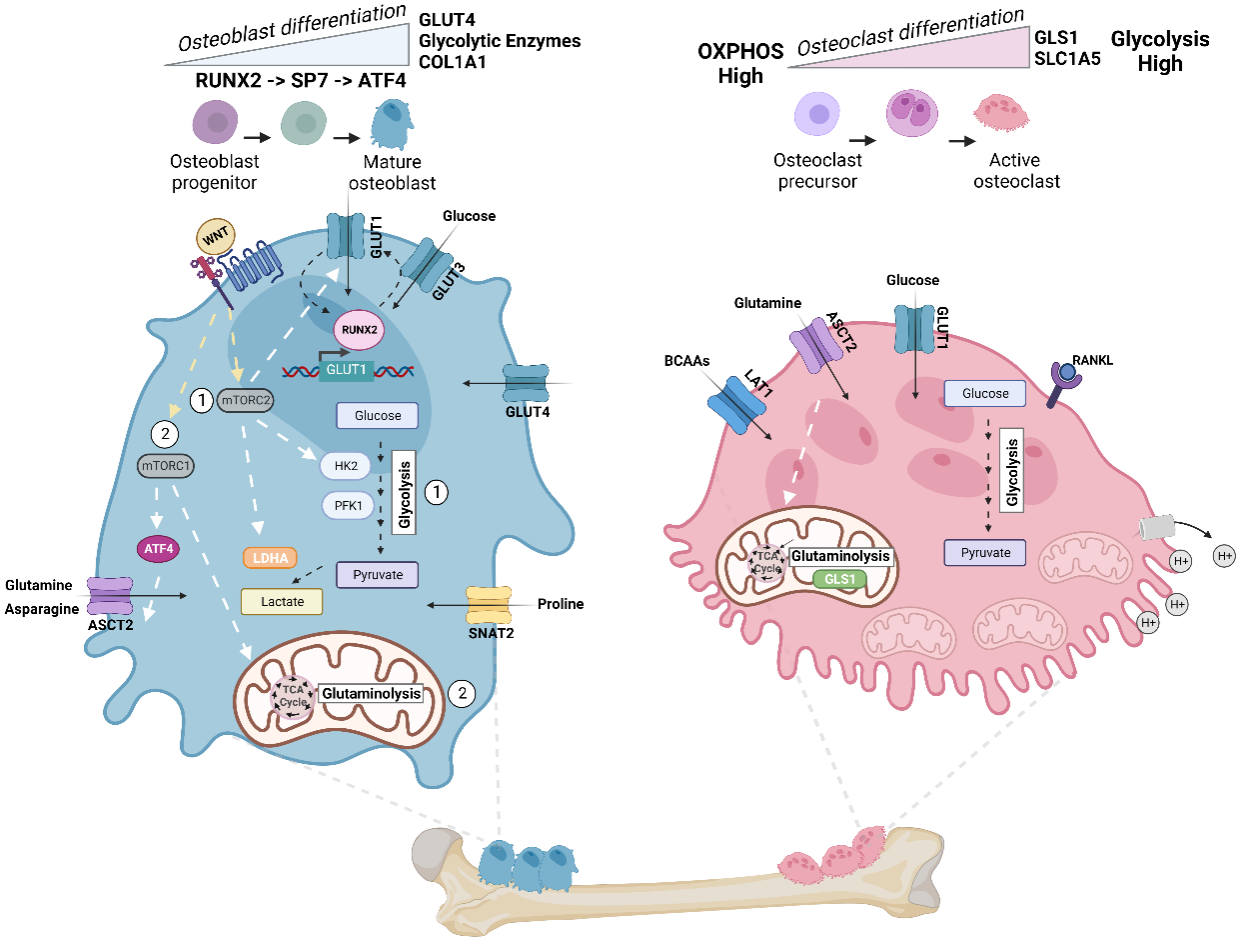
Energy metabolism in osteoblast and osteoclast. Metabolic pathways rewired in osteoblast (Left) and osteoclast (Right) during cellular differentiation. Left: In mature osteoblasts, glycolysis accounts for 80% of ATP production. Osteoblast precursors sequentially express the transcription factor RUNX2, followed by Sp7 (osterix (Osx)), and collagen-promoting ATF4 at late stages of differentiation. Illustration depicts the feed-forward mechanism where high intercellular glucose inhibits the proteasomal degradation of RUNX2, and RUNX2 increases the levels of *GLUT1* transcription. WNT signaling regulates osteoblast activity and has been shown to (1) activate glycolytic enzymes (HK2 and PFK1), GLUT1, and LDHA via mTORC2 and (2) promote glutaminolysis and TCA cycle activation via mTORC1 and downstream regulation of ATF4 and glutamine transporter ASCT2 (*SLC1A5*). Import of asparagine via ASCT2 and proline via SNAT2 (*SLC38A2*) also facilitates osteoblast differentiation. Right: To dissolve bone and degrade collagen, osteoclasts require the generation of protons (H+) by hydrolyzing ATP. Thus, osteoclasts exhibit a significant demand for ATP and contain a high number of mitochondria. ATP demand is met by upregulating both glycolysis and glutaminolysis, and differentiation increases glycolysis and expression of glumaminase 1 (GLS1) and ASCT2. Mid-late stage osteoclast differentiation is also dependent on increased branched chain amino acid (BCAA) pools. Figure was created with BioRender.com.

Bone formation is an energy-intensive process, and the importance of glucose metabolism in bone and osteoblast lineage cells has long been appreciated from studies of bone explants and isolated bone osteoblastic cells (4–6). Specialized osteoblast cells needed to build bone require the generation of high levels of ATP (23). Osteoblasts express 3 glucose transporters (GLUT1, GLUT3, and GLUT4) to facilitate glucose uptake (37–39). *In vitro* studies, including metabolic tracing with murine calvarial cells, showed that osteoblast differentiation under aerobic conditions increases glucose consumption and lactate accumulation, and that aerobic glycolysis accounts for 80% of ATP production in mature osteoblasts (**Figure 3**) (40, 41). The majority of the remaining 20% of glucose-derived carbon is metabolized in the mitochondria through respiration.

The dependence of osteoblasts on aerobic glycolysis is intimately tied to normal differentiation of these specialized cells from mesenchymal precursors. Osteoblasts differentiate through sequential stages, which are associated with regulated expression of distinct transcription factors and markers. Early osteoblast precursors express the transcription factor RUNX2, while later stages of differentiation are activated by the expression of Sp7, also called osterix (Osx). At late stages of differentiation, mature osteoblasts activate expression of activating transcription factor 4 (ATF4), a master transcriptional regulator of amino acid metabolism and stress-response pathways. ATF4 activation promotes an increase in collagen type I (COL1A1) synthesis, as well as other matrix proteins such as integrin-binding sialoprotein (IBSP) and osteocalcin (OCN) (42, 43) (**Figure 3**). Studies by Wei and colleagues illustrated that a feed forward mechanism exists between the glucose transporter GLUT1 and the osteoblast transcription factor RUNX2 (44). High levels of intracellular glucose inhibit the proteasomal degradation of RUNX2, and RUNX2 increases the levels of GLUT1 by binding to and activating the *Glut1* promoter (**Figure 3**). This promotes the uptake of glucose, which in turn enhances osteoblast differentiation and bone formation. The researchers show that GLUT1 is responsible for the majority of glucose uptake in osteoblasts, and that this enhanced uptake is necessary for osteoblast differentiation (44). During differentiation *in vitro*, GLUT4 expression, but not that of GLUT1 or GLUT3, is upregulated. GLUT4 is further induced by insulin-stimulated glucose uptake, revealing that other glucose transporters can contribute to cellular metabolism that supports osteoblast differentiation (38). Moreover, mechanistic data by Esen and colleagues define an axis where Wnt3a contributes to aerobic glycolysis in osteoblasts *via* an mTORC2-AKT axis downstream of Rac Family Small GTPase 1 (RAC1). In this pathway, WNT-LRP signaling promotes bone formation through the induction of key glycolytic enzymes, including GLUT1, Hexokinase 2 (HK2), Phosphofructokinase-1 (PFK1), and Lactate Dehydrogenase A (LDHA) (**Figure 3**) (45). More recent work has also found that, in the presence of glucose, NR2F1, a nuclear hormone receptor and transcription factor, promotes osteoblast survival and osteogenic differentiation *via* AKT/ERK signaling; while in the absence of glucose, expression of NR2F1 is reduced, and activation of this pathway is inhibited (46)

Glutamine serves as an additional fuel source for osteoblasts during osteogenesis (47). There, glutamine is oxidized in the TCA cycle, which contributes to energy production in the mitochondria in osteoblast precursors (48). Indeed, Karner et al. demonstrated that glutamine is required for osteoblast function in response to WNT signaling (48). They show using cultures of osteoblast precursors and *in vivo* mouse models that WNT, *via* mTORC1, stimulates catabolism of glutamine in the TCA cycle, which lowers intracellular glutamine and activates the general control nonderepressible 2 (GCN2)-mediated integrated stress response (ISR) (**Figure 3**). This WNT-mTORC1-GCN2 axis was found to be important for maintaining protein translation during the process of bone anabolism by activating ATF4, which promotes the transcription of genes important for amino acid uptake and biosynthesis (48). More recently, Shen and colleagues found that ASCT2 (*SLC1A5*) is a target of ATF4, downstream of mTORC1, and is the primary transporter of glutamine in response to WNT in osteoblasts (49). Building on this knowledge, Sharma et al. further found, using genetic and metabolic techniques, that SLC1A5 is required for osteoblast proliferation, differentiation, and extracellular matrix synthesis by facilitating the import of glutamine and, to a lesser extent, asparagine for *de novo* non-essential amino acid synthesis and protein production (50). Aside from glutamine and asparagine, osteoblast differentiation increases import of the amino acid proline *via* the neutral amino acid transporter, SNAT2 (*SLC38A2*), thereby facilitating the incorporation of proline into proline-rich osteoblast proteins, such as RUNX2, OSX, OCN, and COL1A1 (51).

### 3.3 Energy metabolism of the osteoclast

Osteoclasts are highly motile cells. Their differentiation, as well as their function in bone resorption, are ATP-demanding processes. As such, osteoclasts have more mitochondria per surface area than virtually any other cell type to support the need for high levels of ATP (52). The function of osteoclasts in dissolving bone and degrading collagen requires the generation of protons (H+) by hydrolyzing ATP in order to support acidification and bone resorption (**Figure 3**) (23). Like osteoblasts, osteoclasts require an upregulation of glycolysis and increase in glucose uptake *via* GLUT1 for their differentiation and function. Depriving osteoclasts of glucose inhibits their bone-resorbing activity (53).

Furthermore, osteoclast differentiation was also found to be dependent on glutamine, as expression of the glutamine transporter *SLC1A5* and the first enzyme in the breakdown of glutamine, glutaminase 1 (*GLS1*), increased during differentiation (**Figure 3**). Depletion of glutamine or inhibition of ASCT2 repressed osteoclast differentiation and function (53). The regulation of glycolysis in osteoclasts is under the control of HIF1a, while c-MYC regulates expression of *SLC1A5* and *GLS1* (53, 54). More recently, Ozaki et al. demonstrated that, aside from glutamine, osteoclast differentiation is negatively regulated by the neutral amino acid transporter, LAT1 (*SLC7a5*) in mice (55). Further, all three BCAAs, particularly valine, as well as the enzymatic activity of branched-chain aminotransferase 1 (BCAT1) are increased during RANKL-induced mid-late stage osteoclast differentiation (56). Demonstrating a complex relationship between the maintenance of amino acid pools, osteoclastogenesis, and bone homeostasis.

Consistent with prior work, Li et al. demonstrate that osteoclasts require both glycolysis and oxidative phosphorylation for proper function (57). However, metabolic dependencies differ between osteoclast differentiation and active bone resorption. As a notable example, it has been shown that the formation of osteoclasts requires OXPHOS, while bone resorption exhibits a greater dependence on glycolysis (**Figure 3**) (52).

### 3.4 ATF4 plays a central role in physiologic bone remodeling

Although studies surrounding the bioenergetics of normal bone homeostasis have been relatively limited, the importance of glucose and glutamine utilization by osteoblasts and osteoclasts has been established. As detailed above, osteoblasts demonstrate a dependence on aerobic glycolysis that is also observed in cancer cells, and both osteoblasts and osteoclasts have a preferential need for upregulating glycolysis for cell differentiation and function. While there is logic to the role of aerobic glycolysis in cellular function, the reasons for this phenomenon have not been fully elucidated in the context of differentiation and require further study. One can speculate that this may be a result of a dependency on glycolytic intermediates to fuel biosynthetic reactions, such as *de novo* amino acid synthesis. Furthermore, the role of other metabolic pathways in osteoclast and osteoblast differentiation and function also remains unanswered. One notable example is the role of ATF4. Osteoblasts upregulate expression of ATF4 in the terminal differentiation process (42). ATF4 has been shown to regulate osteogenic genes and its phosphorylation by RSK2 is needed for differentiation (58). ATF4 enhances bone formation by activating amino acid import and collagen synthesis, and studies have shown that implementing a high-protein diet in mouse models can overcome the effect of ATF4 loss on skeletal formation (58, 59). ATF4 expression in osteoblasts has been demonstrated to be regulated, at least in part, *via* an mTORC1-GCN2-ATF4 axis (48, 60). Interestingly, mTORC1 has been shown to play a role in the ATF4-dependent regulation of serine and glycine biosynthesis and downstream collagen production (61), suggesting another downstream pathway that would explain osteoblast dependence on ATF4 signaling.

## 4 Malignant bone metabolism

### 4.1 Primary bone malignancies

#### 4.1.1 Osteosarcoma

Osteosarcomas (OS)s are the most common primary malignant bone sarcomas, representing 40-50% of bone tumors (62). OS is characterized by a bimodal age distribution with peak incidence in children and adolescents, and a median age of 18, as well as a smaller second peak incidence in individuals over 60 years of age. The worldwide incidence of OS is 1-3 cases annually per million individuals (63). Over 75% of OS cases occur in the metaphysis of the long bones, most commonly in the distal femur, proximal tibia, and humerus (64). The current standard of care for OS includes a combination of chemotherapy, which may include methotrexate, cisplatin, doxorubicin, ifosfamide, and etoposide, as well as surgery. OS cells are not readily responsive to radiation, so it does not play a major role in treatment (65). Due to limited success of new biologically targeted therapies, chemotherapeutic agents have been part of OS treatment for decades, and the standard of care has largely been unchanged. Commonly occurring genetic lesions or pathway alterations have not been identified in OS, though mutations in p53 and Rb have been observed along with significant cases of aneuploidy (66). However, to this date, no clear molecular signatures have been identified that have led to successful targeted therapies (64).

#### 4.1.2 Ewing sarcoma

Ewing sarcoma (ES) is the second most common pediatric bone tumor, with an incidence of 1 case per 1.5 million in the population and presents most commonly during adolescence. ES can arise both in the bone and in soft tissue sites, most commonly in the pelvis and proximal long bones of the body (67). Current treatment regimens include chemotherapy consisting of doxorubicin, etoposide, cyclophosphamide, vincristine, and ifosfamide, as well as local control with surgery and/or radiation (67). ES is characterized by the presence of EWS::ETS fusion proteins, most commonly EWS::FLI1, which results from a chromosomal t(11;22) translocation (67). These fusion oncogenes act as the tumor-initiating event, leading to malignant transformation. EWS::FLI1 acts as an aberrant transcription factor that promotes widespread epigenetic and transcriptional reprogramming through its function at enhancers and gene promoters (68–70). Aside from the presence of the EWS::FLI1 fusion protein, ES tumors are otherwise genomically quiet, with few other recurrent mutations (71–73). Thus far, efforts to target EWS::FLI1 itself have been largely unsuccessful due to its lack of enzymatic activity and disordered structure (67). Thus far, efforts to target EWS : FLI1 itself have been largely unsuccessful due to its lack of enzymatic activity and disordered structure.

Given the lack of therapeutically targetable mutations in primary bone sarcomas, characterization of metabolic dependencies may identify opportunities for novel therapeutic strategies. To date, investigation of the metabolic properties of sarcomas has been limited, but early studies have begun to provide insights. Studies of sarcoma patients identified that their tumors displayed high rates of glycolytic activity and lactate production (74). As a result, fluorodeoxyglucose positron emission tomography (FDG–PET) imaging is used clinically as a staging tool for several diverse types of cancers, including OS and ES, where it can be used to identify tumors at the primary site and skeletal and lymph node metastases. This radiographic tool can in some cases also be used to follow disease progression and to predict patient outcome (75, 76). More recently, data have emerged around the rewiring of other metabolic pathways in sarcomas. In the following sections we will focus on and summarize the current understanding of amino acid pathways dependencies in OS and ES.

### 4.2 Amino acid dependencies in osteosarcoma and Ewing sarcoma

#### 4.2.1 The serine biosynthetic pathway as a key dependency

The most extensively studied amino acid biosynthetic pathway in sarcomas is the SBP. As a non-essential amino acid, serine can be taken up extracellularly or synthesized *de novo*. The SBP utilizes the 3-phosphoglycerate (3-PG) intermediate of glycolysis as the carbon backbone to generate serine *de novo via* three enzymatic steps carried out by the enzymes PHGDH, phosphoserine aminotransferase (PSAT1), and phosphoserine phosphatase (PSPH) (**Figure 1**). PHGDH expression is high in both OS and ES (77–81), and high PHGDH correlates with poor survival in both of these cancers, suggesting a clear dependency on the SBP. The SBP contributes to a broad metabolic hub. In addition to its proteinogenic role, serine has numerous additional biological and metabolic functions. For example, the conversion of serine to glycine by serine hydroxymethyltransferase (SHMT) (isozymes SHMT1, cytosol; and SHMT2, mitochondria) generates one-carbon units that enter two main pathways: the folate cycle and the methionine cycle. These pathways are important for nucleotide synthesis and the generation of the methyl donor S-Adenosyl Methionine (SAM), respectively (**Figure 1**). Glycine and cysteine, derived from the transsulfuration pathway, are also utilized to synthesize GSH. Thus, the sum of metabolites produced by these interconnected networks contribute to *de novo* purine and pyrimidine synthesis, maintenance of GSH and NADPH pools for redox homeosis, as well as the production of methyl groups to fuel DNA and histone methylation reactions (82). Preferential use of the SBP allows cancer cells to take advantage of the pathway for the generation of biomass and to promote cell growth and division.

Methotrexate is a dihydrofolate reductase (DHFR) inhibitor (**Figure 1**) and has been part of the standard of care for OS for over 40 years. DHFR is a critical enzyme in the folate cycle, downstream of the SBP, which catalyzes the reduction of dihydrofolate to tetrahydrofolate (THF), important for DNA synthesis and methylation. These recent mechanistic insights on the role of the SBP and interrelated pathways provide clarity on longstanding empirically determined chemotherapy regimens utilizing methotrexate.

**Figure 4:**
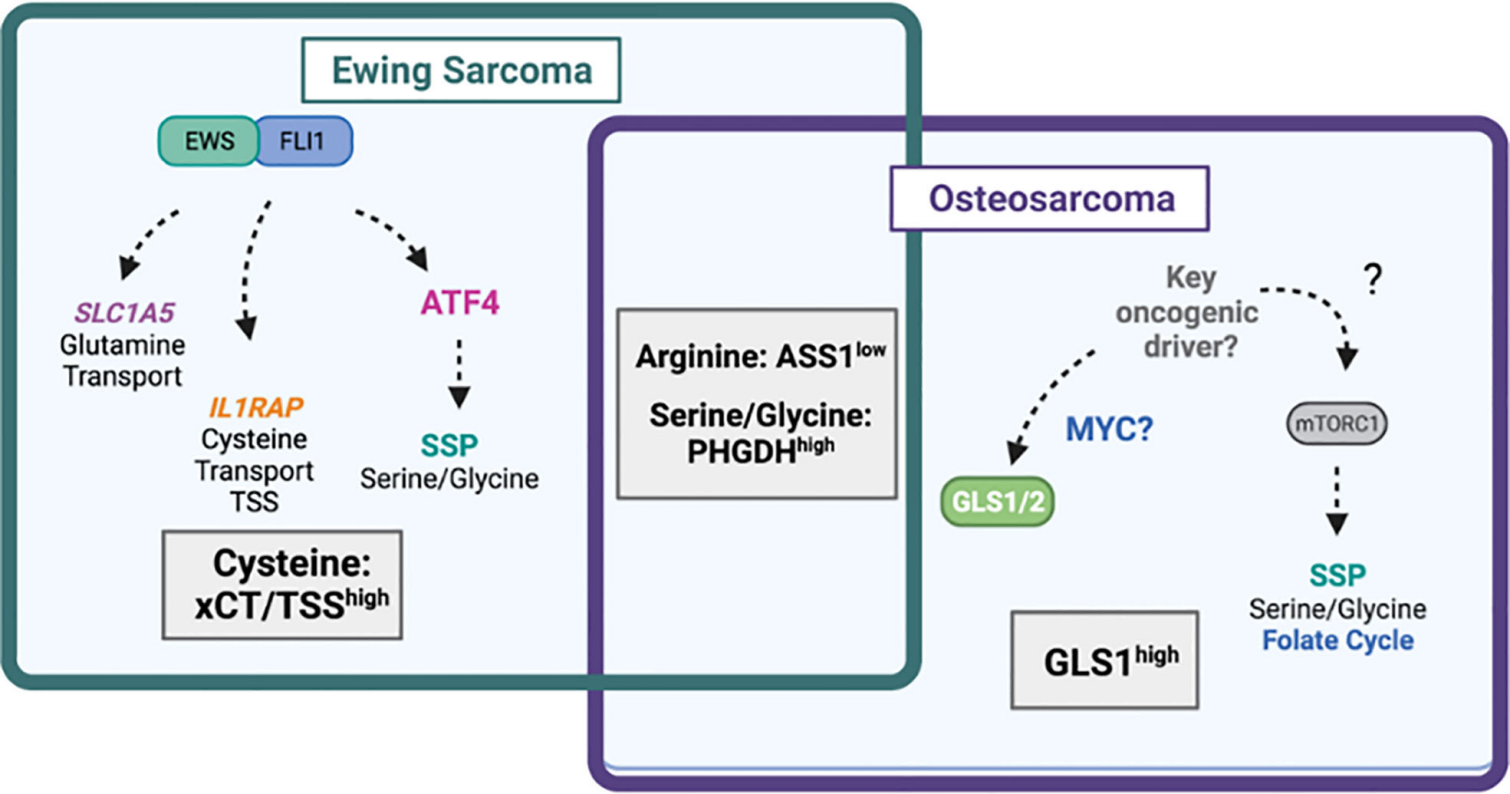
Amino acid dependencies in Ewing sarcoma and osteosarcoma. Venn-diagram depicting shared and unique amino acid vulnerabilities in Ewing sarcoma and osteosarcoma. The EWS::FLI1 fusion protein has been shown to directly regulate expression of glutamine transporter *SLC1A5* (ASCT2) leading to high glutamine import. EWS::FLI1 also regulates expression of *IL1RAP* and downstream cysteine availability via the transsulfuration pathway (TSS) and xCT (*SLC7A11*). EWS::FLI1 indirectly regulates the serine biosynthesis pathway (SSP) via ATF4. In osteosarcoma, the SSP also drives tumorgenicity through undefined mechanisms likely involving mTORC1. The mitochondrial enzyme GLS1, which may be under the control of MYC is highly expressed in osteosarcoma and predicts worse survival. Diagram was created with BioRender.com.

In primary bone sarcomas, the SBP has received extensive attention, given the high levels of PHGDH that are observed in both OS and ES (77–81, 83). Rathore et al. recently explored the effect of PHGDH inhibition in OS cell lines and xenograft models and provided preclinical evidence for the combination of PHGDH inhibition with inhibition of mTORC1. Using metabolomic and lipidomic profiling, the authors identified the accumulation of fatty acids, BCAAs, SAM, and methionine in OS following inhibition of PHGDH using the inhibitor NCT-503 (81). They were able to show that PHGDH inhibition led to increased expression of *SLC7A5* (LAT1) and *SLC3A2* (CD98) transporters that drive transport of leucine into the lysosome, leading to mTORC1 activation (**Figure 4**). Given this finding, the researchers then demonstrated that combining NCT-503 with non-rapalog mTORC1 inhibitor perhexiline led to tumor reduction in xenograft models (81). These studies are consistent with prior work, which also demonstrated that mTORC1 regulates OS cell proliferation partly *via* the regulation of serine/glycine metabolism (83).

Numerous studies have focused on defining the role of the SBP in ES (77–80). This work has shown that PHGDH is overexpressed in ES cell lines and primary patient tumors, and that ES cells are highly dependent on the SBP. Furthermore, several groups, including our own, have demonstrated that the EWS : FLI1 fusion oncogene regulates expression of key enzymes in the SBP, *PHGDH*, *PSAT1*, and *PSPH*, as well as their metabolic products (77, 78, 80, 84).

In cancers without amplification of the *PHGDH* locus, such as lung cancer, activation of the SBP can be mediated through upregulation of ATF4 (85–88). Studies from our lab showed that EWS : FLI1 is able to transcriptionally regulate expression of *ATF4* through direct promoter binding, and that ATF4 expression can also be regulated by the scaffolding protein menin, which also has an oncogenic role in ES (**Figure 4**) (84, 89). However, studies have been inconsistent on the predominate function of the SBP is in supporting ES tumorgenicity. Some evidence suggests that inhibition of the SBP in ES affects redox homeostasis and leads to the accumulation of ROS and DNA damage (78). Other work has implicated PHGDH loss in decreasing 2-hydroxyglutarate levels, increasing total histone-3 levels, and enhancing methylation of histone H3 at lysine (K) 9 and K27. It should be noted that responses varied among the cell lines evaluated (77). These studies suggest that the SBP may serve an oncogenic role in ES by influencing multiple downstream metabolic programs. In either case, recent work by Issaq and colleagues showed that PHGDH inhibition is a targetable vulnerability, where combination with inhibition of nicotinamide phosphoribosyltransferase (NAMPT), which blocks synthesis of the PHGDH substrate NAD+, had a synergistic effect *in vitro* and in *in vivo* xenograft models (80).

While the mechanism of SBP activity in ES can be attributed to the direct and indirect effects EWS : FLI1 fusion-mediated metabolic reprogramming, the mechanism by which the SBP is activated in OS is much less clear, likely due to the lack of known oncogenic drivers. Wildtype p53 has been shown to reduce PHGDH in melanoma (90). The majority of OSs exhibit mutations in p53, suggesting a potential mechanism of SBP regulation (91). Importantly, PHGDH inhibition has been reported to induce cell death in ES (77), but not in OS where PHGDH inhibition resulted in cytostasis (81). Whether this represents unique difference between the tumor types or technical experimental variables requires further study. Further, ES cell lines exhibit varying sensitivities to serine/glycine withdrawal (79), while uniformly showing reduced growth with genetic PHGDH loss of function. Additionally, the effectiveness of PHGDH inhibition in OS cells does not increase their sensitivity to serine/glycine depletion (81). This suggests that ES and OS cells may be more dependent on the *de novo* synthesis of serine than the extracellular import of the amino acid.

#### 4.2.2 Glutamine—a conditionally essential amino acid

Although glutamine is a non-essential amino acid, many tumors rely on extracellular glutamine for survival, therefore classifying it as a conditionally essential amino acid (14). As the most abundant amino acid in the plasma, glutamine is found at levels between 500 and 750µM. In addition to its proteinogenic functions, glutamine also participates in the synthesis of other non-essential amino acids, as well as acting as an essential carbon and nitrogen donor for the synthesis of nucleotides, GSH, and glycosylation precursors.

Numerous studies have identified a dependence of OS and ES cells on glutamine and glutaminolysis. In cancer cells, the rate-limiting enzyme for glutaminolysis is GLS, the mitochondrial enzyme responsible for the conversion of glutamine to glutamate, which can exist as one of two isoforms, GLS1 and GLS2. Molecularly, research has shown that MYC-induced glutaminolysis occurs specifically in MYC-dependent OS cells, but not in osteocytes (**Figure 4**) (92). Work in ES has demonstrated that EWS::FLI1 positively regulates expression of the glutamine transporter ASCT2 (*SLC1A5*), potentially *via* the direct binding of the fusion to a regulatory sequence in the *SLC1A5* gene (**Figure 4**) (78). Clinical studies with OS patients have shown that GLS1 is highly expressed in OS tumor tissue, which predicts poor survival (**Figure 4**). The effect of GLS1 inhibition was recently evaluated in OS cell lines using metabolic flux analysis and tracing studies. These studies demonstrated that pharmacologic inhibition of GLS1 with CB-839, combined with electron transport chain (ETC) inhibitor metformin, reduced primary tumor growth and metastasis by inhibiting glycolytic and TCA cycle activity (93). Furthermore, GLS1 expression decreases with neoadjuvant chemotherapy, suggesting that GLS1 may be predictive of OS treatment response (94). Recent studies have also demonstrated success in therapeutically targeting GLS in cell line and murine models of soft tissue sarcomas (95).

#### 4.2.3 Arginine—a case of auxotrophy in sarcomas

Arginine can be synthesized *de novo* by the action of the argininosuccinate synthetase 1 (ASS1) enzyme, which converts citrulline and aspartate to arginine (**Figure 1**). Arginine auxotrophy can result from the silencing of ASS1 in several tumor types, including sarcomas (96). In cancers such as lymphomas, bladder cancer, and prostate cancer, diminished ASS1 expression can be due to methylation of the *ASS1* promoter (97). In the case of sarcomas, a 2016 study found that almost 90% of sarcoma tumors have low ASS1 expression; however, *ASS1* promoter methylation is not observed in sarcomas. Specifically, Bean et al. evaluated 701 bone or soft tissue sarcomas by immunohistochemistry analysis for ASS1 expression and found that most primary bone tumors (87.2%, 34/39) and soft tissue tumors (86.4%, 572/662) had low ASS1 expression. 3 of 10 OS tumors evaluated were determined to be ASS1 positive, while 1 of 7 ES tumors evaluated were ASS1 positive (98). By characterizing select ASS1^Low^ cell lines, including OS and ES cell lines, the authors showed that arginine deprivation with pegylated arginine deaminase (ADI-PEG20) led to cytostasis and a dependence on autophagy. Combining ADI-PEG20 with chloroquine, an autophagy inhibitor, led to necroptotic and apoptotic cell death. The authors tested this synthetic lethal targeting strategy *in vivo* using subcutaneous xenografts with MNNG/HOS OS cells and showed that combining ADI-PEG20 with chloroquine led to the greatest reduction in tumor growth, relative to single arm treatments (98).

#### 4.2.4 Transsulfuration pathway and cysteine metabolism

Cysteine is another non-essential amino acid, which can be acquired from the extracellular space through several transport mechanisms or synthesized *de novo* in the transsulfuration pathway from serine and methionine (**Figure 1**). Cysteine is the rate-limiting substrate for the synthesis of GSH, which is critical for the maintenance of cellular redox homeostasis. A drop in GSH can lead to the accumulation of reactive oxygen species (ROS) and/or oxidized membrane phospholipids (lipid ROS). An inability to manage ROS leads to the oxidation of various biomolecules and can culminate in cell death. Thus, cancer cells employ a variety of mechanisms to mitigate ROS burden. As it relates to cysteine, many types of cancers will upregulate *SLC7A11*, which encodes for xCT, the rate-limiting subunit of the cystine-glutamate antiporter system x_C_-. In a recent study, application of 3D cultures with ES cells in proteomic and translatomic screens found that the EWS : FLI1 fusion oncoprotein directly induces expression of system x_C_- and the transsulfuration pathway, *via* induction of *IL1RAP* expression, a cell surface IL-1 receptor co-receptor. The researchers found that IL1RAP directly binds system x_C_- to increase its cystine importing activity, and that EWS::FLI1 regulates *IL1RAP* expression through enhancer activation (**Figure 4**). IL1RAP was found to be highly expressed in ES and important for promoting resistance to *anoikis*, detachment-induced cell death, and metastasis (99). Although little is known about the role of cysteine and GSH metabolism in OS, it has been demonstrated that OS cell are dependent on methionine, suggesting that this may be in part to maintain cysteine levels (100).

## 5 Bone microenvironment and metastasis

### 5.1 Metabolism in the bone tumor microenvironment

The function of non-malignant cells in mesenchymal tumors has been less studied when compared to epithelial cancers of adulthood (101). Additionally, a distinguishing feature of sarcomas is that the distinction between malignant and stromal cells can be unclear, due to the mesenchymal origin of the tumor cells themselves (101). Primary bone sarcomas grow in specialized and complex bone microenvironment that are highly vascularized and contain osteoclasts, osteoblasts, osteocytes, stromal cells (MSCs, fibroblasts), vascular cells (endothelial cells and pericytes), immune cells (macrophages, lymphocytes) (102, 103), and a mineralized extracellular matrix (ECM) (104). This is fertile soil for tumor cells to hijack growth promoting pathways such as cytokines, chemokines, and growth factors in the bone microenvironment (65, 105). Remodeling and resorption of bone is a key feature of primary bone malignancies like OS and ES, leading to an osteolytic bone environment that provides space for growth of the tumor (101, 106, 107).The metabolic crosstalk in and among these cell types, and how it influences and is influenced by tumor cells, are important outstanding questions that are beginning to be addressed.

MSCs have been shown to favorably impact OS progression and contribute to osteolysis (108, 109). Several studies have centered around the role of lactate in promoting an acidic microenvironment. Lactate released by both cancer cells and cells in the TME plays a prominent role in tumor progression, by increasing angiogenesis, motility, and the migration of cancer cells (110). Studies in OS models have demonstrated that lactate derived from MSCs feeds OS (111). MSCs are induced by adjacent OS cells to undergo Warburg metabolism and increase lactate secretion and expression of the lactate exporter monocarboxylate transporter 4 (MCT4). Lactate from MSCs feeds OS, and OS cells import lactate *via* monocarboxylate transporter 1 (MCT1). This indirectly increases mitochondrial biogenesis and promotes the migration of OS cells (111). The mechanisms linking lactate metabolism to mitochondrial biogenesis and cell migration remain exciting avenues for further exploration.

Insights into the metabolic functions of the bone TME can also be gleaned from cancers that metastasize to the bone, such as breast cancer, prostate cancer, and multiple myeloma (112). For example, Pollari et al. show that serine from breast cancer cells in the metastatic bone microenvironment contributes to the formation of bone resorbing human osteoclasts and may contribute to osteolysis (113). Additionally, similar to studies in OS, bone metastatic cancer cells have also been shown to release lactate *via* MCT4, which is taken up by MCT1 in osteoclasts to fuel OXPHOS and collagen resorption (114). In both of these cases, the activity of osteoclasts leads to bone resorption, creating a more fertile metastatic niche. Interestingly, studies in multiple myeloma have shown that tumor cells prevent the differentiation of bone marrow stromal cells into osteoblasts in part by depleting glutamine. Finally, osteoblast differentiation increased expression of *ASNS* and asparagine could rescue osteoblast differentiation after glutamine depletion (115). This examination of amino acid cross talk in the primary tumor of OS and ES is clearly incomplete. Further exploration of this complex interaction with tumor-supporting cells in the bone microenvironment will be central to determining the effectiveness of metabolic therapies and predicting potential resistance mechanisms in sarcomas.

### 5.2 Primary versus metastatic metabolism

The ability to starve cancer cells through dietary restriction and/or the use of targeted therapies has garnered extensive attention recently (22). However, the cancer context in which these therapies are evaluated is key to determining their therapeutic potential. Further, it is evident that the metabolic dependencies of the primary tumor differ from those of metastatic lesions. For instance, in breast cancer models, researchers have demonstrated that lung and brain metastases are more sensitive to targeting of the SBP than the primary tumor site, in part due to SBP-dependent activation of mTORC1 (116–119). In the brain, the low availability of serine and glycine makes brain metastases more sensitive to inhibition of PHGDH (118). More recently, work also utilizing breast cancer models has suggested that heterogeneous or low PHGDH protein expression at the primary tumor site is associated with increased metastatic potential. This study shed light on distinct regulation of PHGDH transcript and protein and demonstrated a non-catalytic role for PHGDH in directly interacting with the glycolytic enzyme phosphofructokinase resulting in aberrant protein glycosylation (120). Though PHGDH expression is high in both OS and ES and drives tumorgenicity, the impact of potential heterogeneity in transcript and protein expression on primary growth and metastatic dissemination has not been explored. A deep understanding of metabolic dependencies in the tumor niche in bone for OS and ES compared to common sites of metastasis, such as the lung, is lacking and warrants further study.

Studies have begun to look at metabolic differences associated with metastasis in bone sarcomas. Using metabolic profiling and metabolic flux analysis in high and low metastatic cell lines, recent work has suggested that metastatic OS cells activate pathways such as arginine, GSH, and fatty acid metabolism (121–123). Additionally, GLS1 has recently been shown to play a role in promoting metastasis in OS (93). Parallel studies in ES have been more limited, though as discussed above, recent work has implicated cysteine metabolism downstream of IL1RAP in promoting metastasis (99). This work would benefit greatly from studies in additional models of spontaneous metastasis, as the current models do not readily metastasize before mice succumb to primary tumor burden (124).

## 6 Therapeutic applications targeting amino acid metabolism in sarcomas

The current standard of care for sarcoma, consisting of surgery, chemotherapy and radiation has improved the life expectancy for patients, but survival has since plateaued without meaningful improvements in 30 years. Therefore, new therapies as well as diagnostic and prognostic tools are needed. As it relates to metabolism, there are new therapies under clinical evaluation for sarcomas. As previously discussed, preclinical studies showed that ~90% of sarcomas have low expression of *ASS1*, making these cells more dependent on extracellular sources of arginine (98). Treatment of ASS1^Low^ sarcoma cell lines with PEGylated arginine deiminase, ADI-PEG20, sensitized cells to cell death induced by the chemotherapeutic agents, gemcitabine and docetaxel. This prompted a Phase II clinical trial that started in May 2018 in which PEGylated arginine deiminase, ADI-PEG 20, was used in combination with gemcitabine and docetaxel for the treatment of bone and soft tissue sarcoma, including OS, ES, and small cell lung cancer (NCT03449901) (98, 125). However, challenges in the clinical use of ADI-PEG20 are likely based on preclinical observations. These may include acquired resistance to ADI-PEG20 by upregulation of ASS1 (98) or rewiring of other metabolic pathways, such an increased dependence on glutamine anaplerosis and the SBP upon arginine starvation (126). Targeting these compensatory pathways with addition of a GLS inhibitor or PHGDH inhibitor has been found to result in synthetic lethality in several models, including leiomyosarcoma (126).

Telaglenastat (CB-839) is a potent orally bioavailable GLS inhibitor that has exhibited anti-tumor activity in breast cancer and lymphoma (127, 128). Current clinical trials are evaluating its efficacy in hematologic and solid cancers (129). A recent metabolomics study tested the effect of GLS inhibition with CB-839 in undifferentiated pleomorphic sarcoma (UPS) and soft tissue sarcomas and found that GLS inhibition causes tumor cell death (95). These findings are promising for the translation of CB-839 to the clinic, especially for OS patients who exhibit high GLS1 activity (93, 94). However, the use of GLS inhibitors needs to be carefully evaluated. In some contexts, GLS dependency may be a phenomenon of cancer cells *in vitro*, where tumors are able to acquire compensatory mechanisms when GLS is inhibited *in vivo* (130, 131).

Given the dependency of numerous cancers on the SBP and PHGDH expression, PHGDH inhibition is also a promising therapeutic vulnerability. In fact, several small molecule enzymatic inhibitors against PHGDH are under preclinical development (116, 132, 133); however, none are yet ready for clinical examination (134).

Metabolomics technology has allowed for the profiling of cancer-associated metabolites, which could provide insight into tumor specific biomarkers. While this has led to progress in identifying cancer-specific prognostic and diagnostic biomarkers in other cancer types (135), there are only a few studies that have focused on sarcoma metabolomics (136). Early work in 2011 used liquid chromatography-tandem mass spectrometry (LC-MS/MS) in formalin-fixed and paraffin-embedded (FFPE) patient tissue from soft tissue sarcomas, including leiomyosarcoma, liposarcoma, and synovial sarcoma, and showed that 8 metabolites exhibited differential abundances between soft tissue sarcoma samples and control tissue (137). Researchers have also investigated liposarcoma cell lines *in vitro*, revealing high consumption of nucleosides and amino acids such as arginine, glutamine, and serine. Through *in vitro* and *in vivo* models, their studies further implicate nucleoside salvage pathway activity as a potential metabolic biomarker predicting response to gemcitabine (138).

More recent work has shifted to metabolomics analysis of patient serum in search of prognostic biomarkers. This work has identified depletion of citrulline, a precursor for arginine synthesis, in high risk metastatic soft tissue sarcoma patients (139). Very few studies have focused on OS and ES patients, specifically. Work by Jia et al. used LC/MS to evaluate changes in plasma amino acid levels between 23 sarcoma patients, including OS and ES, compared to 30 healthy control subjects and identified 4 amino acids or amino acid derivatives (glutamine, sarcosine, homoproline and citrulline) that decreased and 3 (carnosine, lysine, and glutamic acid) that increased in sarcoma patients (140). One of the first metabolic profiling studies in OS utilized LC/MS to investigate the serum and urine of OS patients compared to benign tumor patients and healthy controls and identified down-regulated lipid metabolism and upregulated amino acid metabolism among the pathways impacted (141). Though these patient-centered studies are preliminary, this and future work in this area may provide leads or insights that will subsequentially require further evaluation in larger cohorts of patients to determine the utility of metabolic profiling. The hope is that the identification of bone sarcoma-specific metabolic biomarkers will allow this technology to be used in the future for the diagnosis and monitoring of sarcoma patients (142).

## 7 Conclusions and future directions

In recent years, the exploration of dysregulated amino acid metabolism pathways has garnered increased attention in sarcomas, with exciting new findings on pathways that may serve as tumor-specific vulnerabilities. As discussed above, the potential for basic discoveries in sarcoma metabolism to lead to clinical translation is exemplified by the identification of dysregulated arginine metabolism in bone sarcoma cells and subsequent inclusion of sarcoma patients in a Phase II clinical trial (NCT03449901) that is testing ADI-PEG20 in combination with chemotherapy (98). There is also clear precedent for success including amino acid targeting agents as adjuvant therapies in other cancers. ASNase, which depletes circulating asparagine, has been used in pediatric patients with acute lymphoblastic leukemia since the 1970s. Other amino acid pathways are being evaluated in clinical trials, such as indoleamine-2,3-dioxygenase-1 (IDO1) inhibitors, epacadostat and indoximod, for tryptophan catabolism in cervical cancer and glioblastoma (143–145). Additionally, activation of the SBP has been demonstrated to predict poor prognosis in both ES and OS and to be regulated by an EWS : FLI1-ATF4 axis in ES (77–81, 83). Preclinically, inhibitors of biosynthetic enzymes, such as the SBP enzyme PHGDH are under development for a wide array of cancers (116, 132, 133), and drugs to target amino acid transporters, such as LAT1 (*SLC7A5*), are also being explored (146, 147).

Primary pediatric bone sarcomas arise during a time in development when normal physiologic processes direct bone remodeling that is required for bone growth. This remodeling relies on the activation of transcriptional and metabolic pathways that support high-energy demands, including the increased import and synthesis of amino acids (42, 48). Transcription factors such as ATF4 are activated during osteoblast differentiation in order to maintain amino acid pools and downstream amino acid-dependent products, like collagen (42, 58, 59). Moreover, distinct metabolic programs and dependencies are evident across heterogeneous cell types in the sarcoma tumor niche, including osteoblasts, osteoclasts, and tumor cells, adding to the complexity of dissecting metabolic dependencies in the bone tumor ecosystem. Further, emerging work in sarcoma and other cancer types, has shown that amino acid metabolism differs among distinct tumor cell subpopulations, and that individual metabolic pathways may serve as biomarkers of metastatic potential (93, 99, 119, 120). Intratumor metabolic heterogeneity and differential amino acid requirements among tumor cells, microenvironment components, and between between primary and metastatic sites are poorly understood in the context of primary bone tumors and filling these gaps in knowledge should be a priority for investigation in sarcomas.

Given the dynamic nature of metabolic requirements during bone differentiation, the effect of amino acid targeting therapies on normal developmental processes will require thoughtful consideration, especially in the context of pediatric cancer patients who are still growing. Many other questions will also need to be addressed when investigating metabolic dependencies in the bone tumor niche. For example, the precise cell of origin for both OS and ES is still unclear and highly debated (62, 148), and the timing of genetic and signaling events during development which govern malignant transformation are not well understood. As such, it is not possible to directly compare metabolism in tumor cells to the normal precursor cell from which they arose. Furthermore, how dysregulated metabolism contributes to oncogenesis within cells undergoing transformation in the context of a bone microenvironment is not known. Nevertheless, given recent exciting advances in our understanding of bone and cancer metabolic programs in general, and of distinct amino acid dependencies in OS and ES, the promise of targeting metabolic processes in these tumors is high. Based on precedent with metabolic therapies and dynamic compensatory mechanisms, it is likely that combination approaches and targeting co-vulnerabilities will serve as the most viable option for patients.

## Author contributions

JAJ, EL and CL contributed to the conception and design of the review. JAJ wrote the first draft of the manuscript. All authors contributed to the article and approved the submitted version.

## Funding

Support for this work was provided by the following sources: National Institute of Health: F31 CA254079 (JJ), R01 CA218116 (EL), R37CA237421, R01CA248160, R01CA244931 (CL).

## Conflict of interest

CL has received consulting fees from Astellas Pharmaceuticals, Odyssey Therapeutics, and T-Knife Therapeutics, and is an inventor on patents pertaining to Kras regulated metabolic pathways, redox control pathways in pancreatic cancer, and targeting the GOT1-pathway as a therapeutic approach (US Patent No: 2015126580-A1, 05/07/2015; US Patent No: 20190136238, 05/09/2019; International Patent No: WO2013177426-A2, 04/23/2015).

The remaining authors declare that the research was conducted in the absence of any commercial or financial relationships that could be construed as a potential conflict of interest.

## Publisher’s note

All claims expressed in this article are solely those of the authors and do not necessarily represent those of their affiliated organizations, or those of the publisher, the editors and the reviewers. Any product that may be evaluated in this article, or claim that may be made by its manufacturer, is not guaranteed or endorsed by the publisher.
